# The Impact of Disease X on Potential Travelers’ Travel Decision

**DOI:** 10.3390/ijerph21121607

**Published:** 2024-11-30

**Authors:** Robertico Croes, Jeong-Yeol Park, Kenneth Alexander, Chaithanya Renduchintala, Frank Badu-Baiden

**Affiliations:** 1 Rosen College of Hospitality Management, University of Central Florida, Orlando, FL 32819, USA; jeong-yeol.park@ucf.edu (J.-Y.P.); chait.rendu@ucf.edu (C.R.); frank.badubaiden@ucf.edu (F.B.-B.); 2 College of Medicine, University of Central Florida, Oralndo, FL 32827, USA; kenneth.alexander@ucf.edu

**Keywords:** pandemic, Disease X, speed, spread, severity, travel intention, Orlando, ANCOVA

## Abstract

This study used ANCOVA models to investigate how pandemic characteristics—spreading speed, severity, and vaccination requirements—affect travel intentions. The results reveal that these factors explain 31.7% of the variance in travel decisions, with disease-spreading speed and severity being the most significant determinants. While vaccination requirements are relevant, they play a secondary role compared to the immediate threat of disease characteristics. The interaction effects between these factors further demonstrate their combined impact on travel reluctance. Demographic variables, such as gender and the presence of children, also influence decisions in specific contexts. These findings contribute to the understanding of risk perception during health crises, reinforcing the role of perceived severity in shaping cautious travel behavior. Practical implications for the tourism industry include the need for transparent communication, tailored health protocols, and demographic-specific marketing strategies. Future research should explore broader factors and adopt longitudinal approaches to capture evolving travel intentions during pandemics.

## 1. Introduction

The vulnerability of the tourism sector to pandemics has become increasingly evident, particularly during the COVID-19 pandemic [1,2]. Tourism’s unique characteristics, such as intangibility, heterogeneity, and reliance on advance bookings, create a complex landscape fraught with risks and uncertainties. For example, the time lag between booking and actual travel exposes tourists to dynamic health conditions and regulatory changes. During health emergencies, tourists often face amplified anxiety due to their limited knowledge of unfamiliar destinations [3]. The COVID-19 pandemic has starkly illustrated how these challenges disrupt global travel, with rapid disease transmission and evolving government responses leading to widespread cancellations and revenue losses. The sector’s dependence on human mobility further increases its susceptibility to pandemics, as global connectivity accelerates the spread of infectious diseases. This dual reality—tourism’s economic importance and its heightened exposure to health risks—demands robust research on how pandemics influence traveler behavior and sector resilience.

Despite the substantial attention paid to the pandemic’s impacts on travel, significant gaps remain unaddressed. First, current studies primarily examine known pandemics, such as SARS or COVID-19, providing limited insights into how travelers might respond to hypothetical, yet plausible, threats such as Disease X, an unknown pathogen with epidemic potential [4,5]. Second, much of the existing literature assumes that travelers make rational decisions based on logical evaluations of risks and benefits. However, this assumption overlooks the cognitive and emotional constraints that frequently dictate behavior during health crises [1,6]. Emotional responses, such as fear and anxiety, often dominate decision-making in uncertain situations, leading to behaviors that deviate from traditional economic models. Third, while prior research has analyzed individual pandemic attributes, such as severity or transmission speed, there is limited understanding of how a combination of factors interact to influence travel intentions [2]. Addressing these gaps is crucial for developing a comprehensive framework for understanding travel behavior during pandemics.

This study addresses these gaps by analyzing the impact of five critical pandemic attributes—geographic spread, transmission speed, illness severity, disease movement, and vaccination requirements—on travel intentions during health crises. These attributes were selected for their demonstrated influence on risk perception and decision-making. For example, geographic spread shapes whether travelers perceive a destination as safe, and localized outbreaks may deter fewer tourists than global pandemics [7]. Transmission speed exacerbates uncertainty, as rapid escalation of cases, such as during COVID-19, often leads to last-minute cancellations [1]. Similarly, illness severity, including morbidity and mortality rates, directly affects travelers’ willingness to take risks, with severe diseases creating significant psychological barriers [8]. Meanwhile, vaccination requirements serve as a mitigating factor, restoring confidence and bridging public health efforts with tourism recovery [6]. Unlike context-specific factors, such as quarantine policies or economic costs, these attributes universally affect decision-making by addressing core psychological concerns, making them integral to understanding travel behavior during pandemics.

To investigate how these pandemic attributes influence travelers’ behavior, this study applies a bounded rationality framework. This framework accounts for the cognitive and emotional constraints that shape decision-making in uncertain conditions, offering a more realistic alternative to rational choice models [9]. Unlike rational models, which assume that travelers weigh risks and benefits logically, bounded rationality recognizes that individuals often rely on heuristics, emotional cues, and incomplete information to make decisions. For instance, media coverage, fear of infection, and inconsistent policy enforcement often amplify perceived risks and driving behaviors that might not align with objective realities [1]. During the COVID-19 pandemic, such factors played a significant role in shaping tourist behavior, as fear of infection or quarantine outweighed the logical assessment of low transmission risks in certain destinations [6]. By integrating bounded rationality, this study explores how pandemic attributes interact with psychological responses, providing a nuanced understanding of travelers’ behavior during health crises.

This study aims to bridge the identified gaps by answering three critical questions: (1) How do pandemic attributes, such as geographic spread and transmission speed, shape travelers’ risk perceptions and travel intentions? (2) What socio-demographic and socio-economic factors influence travel decisions during health crises and how do these factors interact with pandemic characteristics? (3) How has the COVID-19 pandemic reshaped travelers’ perceptions of health risks and their long-term travel behavior? Using an experimental design, this study presented participants with hypothetical pandemic scenarios constructed using a 2 × 2 × 2 × 2 factorial design. This approach allows for a controlled investigation of how pandemic attributes interact to influence travel intentions, offering insights into responses to both known and unknown pathogens [4]. By incorporating bounded rationality into the analysis, this study moves beyond traditional models, offering practical implications for policymakers and tourism stakeholders. The findings provide actionable strategies to build resilience, restore traveler confidence, and mitigate the economic impacts of future pandemics, particularly in tourism-dependent regions like Orlando and Florida.

## 2. Literature Review

### 2.1. Pandemic Characteristics, Travel Intentions, and Bounded Rationality

This study differentiates itself from prior research by addressing critical gaps in the understanding of how pandemic characteristics, travel intentions, and bounded rationality intersect to shape travelers’ behavior. While substantial research has focused on known pandemics, such as SARS and COVID-19, these studies offer limited insights into hypothetical threats, such as Disease X, an unknown pathogen with epidemic potential [4,5]. Unlike past research, which largely assumes rational decision-making [10], this study emphasizes the role of bounded rationality, acknowledging the cognitive and emotional constraints that often drive behavior during health crises.

Bounded rationality explains how individuals rely on simplified mental models, heuristics, and emotional cues such as fear or anxiety when processing complex and uncertain pandemic information [11]. For example, emotional responses, such as fear, anxiety, and uncertainty, frequently override rational evaluations, leading to behaviors such as avoidance, over-preparedness, or panic-driven choices when making travel plans [1,6]. By integrating psychological theories, this study highlights the importance of subjective perceptions in travel decision-making, moving beyond traditional economic models. Moving beyond traditional economic models is essential for understanding travel behavior during health crises and pandemics, as such models often overlook the emotional and psychological factors that heavily influence decision-making.

Travelers frequently rely on heuristics and emotional cues, such as fear or anxiety, rather than logical risk assessments, particularly under uncertain conditions. For instance, during the COVID-19 pandemic, widespread fear amplified by media coverage led to the avoidance of entire regions, even when the actual risk was localized [6]. Similarly, perceived risks of fast-spreading illnesses or high-severity outbreaks, such as Ebola, often result in exaggerated avoidance behaviors driven more by emotional responses than objective evaluations of danger [12]. Trust in institutions and public health measures, such as vaccination policies, further shapes travel intentions, with some travelers feeling reassured, while others, influenced by skepticism or misinformation, remain hesitant [13]. Incorporating psychological frameworks, such as bounded rationality, into tourism research is critical to addressing these gaps, enabling policymakers to design interventions that resonate with both the cognitive and emotional dimensions of traveler behavior [11].

Pandemic characteristics, such as geographic spread, transmission speed, severity of illness, and vaccination requirements, significantly influence travel intentions, but their effects are deeply intertwined with bounded rationality. For example, geographic spread often leads to generalized avoidance behaviors because travelers overestimate risks in peripheral areas. This reaction, driven by heuristics and emotional responses, was evident during the COVID-19 pandemic when entire regions were avoided despite localized outbreaks [6]. Similarly, transmission speed intensifies uncertainty, prompting decisions based on fear of rapid contagion rather than personal exposure risk. Media amplification of fast-spreading diseases can overshadow logical assessments, as seen in the widespread cancellations during COVID-19’s early phases [10]. The severity of the illness further compounds emotional reactions, with heightened mortality fears triggering exaggerated risk perceptions, as observed during the Ebola outbreak [14]. Vaccination requirements, while potentially mitigating fears, are also subject to bounded rationality, as trust in health systems and emotional reactions to vaccine policies influence their efficacy in restoring travel confidence [13].

The interaction between these characteristics magnifies their individual effects on travel intentions. For instance, a fast-spreading, severe illness may provoke more profound avoidance behaviors, while vaccination requirements may either reassure or fail to mitigate fears, depending on travelers’ cognitive biases. This dynamic interplay underscores the necessity of understanding travel decisions not as purely rational reactions but as outcomes shaped by subjective perceptions and bounded rationality.

### 2.2. The Research Model

**Figure 1:**
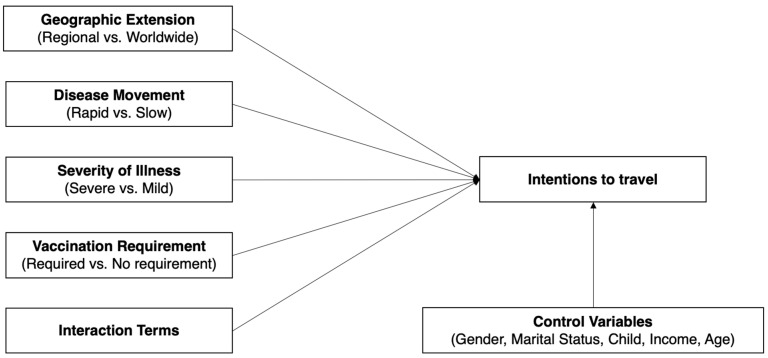
The research model.

The research model illustrated in Figure 1 serves as the backbone of this study, providing a robust framework for investigating the complex interplay between pandemic characteristics and travel intentions. This model is not only conceptually sound but also methodologically rigorous, as it integrates expert knowledge and empirical data to yield reliable insights into how health crises influence travel behavior. The significance of this approach lies in its potential to inform effective strategies for the tourism industry, helping stakeholders navigate the uncertainties posed by future health emergencies.

Central to the model was the systematic assessment of key pandemic-related attributes (Table 1). Each attribute represents a binary decision point encapsulating critical pandemic characteristics: geographic extension, disease movement, severity of illness, speed of spread, and vaccination requirements. The selection of these attributes was deliberate and informed by a literature review and discussions with experts in virology and epidemiology, ensuring that they reflect real-world factors that could influence travelers’ intentions. This evidence-based selection enhances the relevance and applicability of the research findings, providing a clearer understanding of how these pandemic characteristics can shape consumer behavior.

The design allows for precise and systematic manipulation of these attributes within an experimental framework, a methodology well-established in behavioral research. Such controlled experimental designs have been recognized for their effectiveness in capturing decision-making processes in various fields, including marketing and consumer behavior [15]. By employing this method, the study isolates the specific impact of each characteristic on travel intentions, providing granular insights essential for understanding the nuances of traveler behavior during pandemics.

Moreover, the comprehensive design of the model addresses the critical need for a nuanced understanding of travel intentions in the context of health crises. As the tourism industry grapples with the lingering effects of the COVID-19 pandemic, there is an urgent demand for research that elucidates how various pandemic-related factors influence consumer choice. Previous studies have highlighted the importance of perceived health risks in shaping travel intentions [16,17], but this research takes a significant step further by systematically analyzing multiple pandemic characteristics in a controlled experimental setting.

### 2.3. Hypotheses Development

The application of bounded rationality as a theoretical framework provides valuable depth in understanding how travelers process information and make decisions during a pandemic. According to [11], bounded rationality posits that individuals do not make decisions based on complete or optimal information. Instead, they simplify complex decision-making processes using cognitive shortcuts or heuristics, influenced by their emotional responses and limited cognitive capacity. In the context of pandemic-related travel, this theory is especially pertinent, as travelers face heightened uncertainty with limited access to accurate, timely information about health risks, government regulations, and local safety measures. This creates an environment where travelers rely on subjective perceptions, often distorting their judgments through biases such as risk aversion or overestimating potential threats, as highlighted by [1,14].

During pandemics, uncertainty is amplified by the intangibility of travel decisions, as tourists often lack direct experience with a destination or firsthand information on public health risks. This lack of certainty, combined with significant time lags between booking and actual travel, makes tourists particularly vulnerable to external influences that can shape their perceptions of risk and safety. Studies like [3,9] emphasize how these uncertainties increase the reliance on simple heuristics, such as news media coverage, government advisories, and peer opinions. For instance, travelers may prioritize emotional cues or social influences over objective data, leading them to delay trips to low-risk destinations or proceed with travel to high-risk areas based on perceived safety. By employing bounded rationality, this study challenges the traditional assumption of fully rational decision-making in tourism research [2,6], offering a more nuanced understanding of how psychological, emotional, and informational constraints shape travel behavior in the face of a pandemic. This framework not only provides a clearer picture of how tourists navigate uncertainty but also informs strategies for tourism stakeholders, particularly in high-demand destinations such as Orlando and Florida, where understanding these decision-making processes is crucial for effective crisis management and recovery.

Pandemics introduce new variables such as perceived risk and safety into the decision-making process. Research shows that pandemic characteristics, such as infection risks, government-imposed restrictions, and public-health protocols, strongly influence travel behavior [6]. The World Health Organization (WHO) identifies five key characteristics of disease outbreaks that affect travelers’ perceptions: geographic spread, disease movement, severity of illness, transmission speed, and vaccination requirements. These variables not only diminish tourism demand but also threaten the economic recovery of tourism-dependent destinations during health crises [5].

The literature highlights the critical role of risk perception in travel behavior. For example, geographically contained outbreaks such as SARS, MERS, and Ebola boosted travel confidence, as tourists felt safer traveling to affected but controlled regions. In contrast, the global spread of COVID-19, with its rapid transmission and interconnectedness, caused a sharp decline in travel intentions [4]. Research also shows that travelers prioritize disease severity and transmission speed when making decisions during pandemics, because these factors significantly influence their perceived level of risk [1].

Interactions among pandemic characteristics further amplify their effects on travel behavior. Studies suggest that severe illness combined with rapid transmission significantly heightens negative risk perceptions, deterring travel more strongly than either factor alone [18,19,20]. While vaccination requirements are important, they are often perceived as secondary concerns compared to immediate risks, such as disease spread and severity [21]. Consequently, travelers may prioritize destinations where public health measures, including vaccination, align with their safety perceptions.

This study systematically examined the effects of these variables and their interactions on travel intentions through an experimental framework. The model allows for the controlled manipulation of key pandemic characteristics to assess their influence on travel intentions, offering critical insights into how individual and combined factors shape travel behavior under varying conditions of perceived risk. Based on the literature and the theoretical framework of bounded rationality, we propose the following hypotheses:

**H1.**  *Increased pandemic risks, such as widespread geographic spread, rapid transmission, and severe illness, will lead to a decrease in travel intentions.*

**H2.**  *Geographic containment of a disease (e.g., SARS, MERS, Ebola) will result in higher travel intentions compared to globally widespread pandemics, such as COVID-19.*

**H3.**  *The interaction between high disease severity and rapid transmission amplifies their negative effects on travel intentions.*

**H4.**  *Vaccination requirements will moderately influence travel intentions, with their impact being secondary to more immediate perceived risks, such as transmission speed and severity.*

These hypotheses form the basis for a detailed exploration of how pandemic characteristics influence travel behavior. These findings provide actionable insights for policymakers and the tourism industry to better mitigate the negative impacts of future outbreaks on tourism demand.

## 3. Materials and Methods

### 3.1. Survey Instrument and Data Collection

The survey instrument for this study was designed to comprehensively capture factors influencing travel intentions during a pandemic. It included key sections such as demographics, previous travel experience, trip characteristics, health vulnerability, and travel intentions. The scales for measuring prior travel experience and trip characteristics were adapted from the framework developed by [22], ensuring construct reliability and validity. Travel intention items were based on the framework developed by [1], providing a robust measure of how individuals plan or anticipate travel during health crises.

Collaborating with virologists and epidemiologists from the University of Central Florida, the research prioritized pandemic-related attributes such as geographic spread, disease movement, illness severity, rapidity of transmission, and vaccination requirements. These attributes were grounded in empirical evidence and expert consensus, ensuring their relevance and scientific validity. This approach enhanced the robustness of the survey instrument, ensuring that the attributes under analysis were both scientifically sound and practically significant in the context of travel decisions during a pandemic.

Orlando, Florida, was selected as the focus of this case study due to its unique position as a global tourism hub. The city is home to some of the world’s most visited theme parks and attractions, such as Walt Disney World and Universal Studios, making it highly dependent on tourism revenue. In 2022, Orlando welcomed 74 million visitors, maintaining its leadership position as America’s most-visited destination [23]. As a result, travel-related decisions in the context of a pandemic have significant economic and social implications for the region. Additionally, Orlando attracts a diverse range of visitors, both domestically and internationally, providing a rich context for understanding how pandemic-related factors influence travel intentions. The region’s reliance on leisure tourism, rather than business or essential travel, further underscores its relevance as a setting for exploring the discretionary nature of travel decisions during public health crises. The economic impact of Orlando’s tourism industry was estimated at USD 87.6 billion in 2022, highlighting the city’s dependence on leisure tourism [24].

Participants were recruited via Prolific, targeting individuals residing in the United States who had visited Orlando in the past and were planning to return within the next year. This targeted sampling strategy controlled for prior familiarity with the destination, allowing for more nuanced insights into how pandemic risks influence travel decisions. Participants were explicitly informed that the survey involved hypothetical scenarios, and their responses were framed as reflective of intentions rather than real-world constraints. After removing incomplete responses, the final sample consisted of 735 valid responses.

### 3.2. Scenarios

This study examined how information about a viral disease influences travelers’ intentions to visit Orlando, Florida. To ensure the plausibility and relevance of the scenarios, they were developed in consultation with virologists and epidemiologists from the University of Central Florida. Their expertise guided the systematic variation of four pandemic-related attributes: geographic spread, transmission speed, illness severity, and vaccination requirements. This collaborative process bolstered the scientific rigor of the survey, aligning it closely with the study’s objectives and enhancing its practical applicability.

This study employed a factorial design (2 × 2 × 2 × 2) to systematically assess these factors across 16 scenarios. Essentially, the factorial design included a 2_Spreading Speed: Fast vs. Slow_ × 2_Severity of illness: Severe vs. Minimal_ × 2_Vaccination requirement: Yes vs. No_ × 2_Geographic spread: US only vs. Worldwide_. Participants were randomly assigned to one of the 16 scenarios to ensure unbiased responses and even distribution across the sample. They were explicitly informed at the outset that the survey involved hypothetical scenarios, framing their responses as reflective of intentions rather than constrained by real-world limitations.

**Figure 2:**
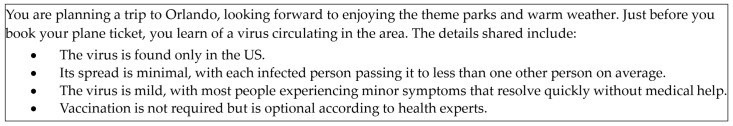
Example scenario.

Travel intention was measured using a seven-point Likert scale (1 = Least likely, 7 = Most likely). The flexibility of the Likert scale allowed participants to express a range of responses, addressing concerns that a binary decision model would oversimplify the decision-making process. This approach captured the complexity and variability inherent in travel-related decisions during a pandemic. An example of a scenario provided to participants is shown in Figure 2.

### 3.3. Analysis Methods

This research utilized a series of Analysis of Covariance (ANCOVA) models to analyze how specific pandemic-related factors—namely, disease spread speed, severity, and vaccination requirements—impact individuals’ travel intentions. ANCOVA was chosen because it allows for the control of confounding variables, such as demographic characteristics (e.g., age, gender) and prior travel behavior, which might otherwise influence travel decisions. By accounting for these covariates, ANCOVA isolated the effects of the independent variables (e.g., spread speed, severity) on travel intentions, offering a clearer and more accurate understanding of their impacts. This approach also reduced error variance, improving precision and enhancing the ability to detect significant differences.

The analysis was conducted using three distinct models to examine the influence of virus attributes under varying contexts. The first model, an overall analysis, included all three factors—spread speed, severity, and vaccination status—across the entire sample, without distinguishing between local and global disease contexts. The second model focused on localized perceptions, analyzing scenarios where the outbreak was confined to the United States to assess how localized disease spread influenced travel behavior. The third model examined global perceptions, investigating how scenarios involving international disease spread impacted travel decisions. Higher-order interactions, such as four-way interactions, were excluded to avoid unnecessary complexity, which could obscure meaningful patterns. This decision aligns with recommendations from [25,26], ensuring that the analysis remained interpretable and yielded actionable insights into how pandemic characteristics shape travel intentions.

## 4. Results

### 4.1. Socio-Demographic Information of the Sample

Table 2 summarizes the socio-demographic characteristics of the 735 respondents. Gender distribution showed 51.43% of respondents identified as female, 47.35% as male, with small percentages identifying as non-binary/third gender (0.54%) or preferring not to disclose their gender (0.68%). Age groups were well-distributed, with the largest proportion in their 50s (22.72%), followed by respondents in their 20s (18.64%), 30s (17.01%), and 60s (14.15%).

Marital status revealed that 44.08% of respondents were married, while 39.32% had never married. Smaller proportions reported being divorced (11.70%), separated (1.90%), or widowed (1.77%). Income distribution was also diverse, with the most common bracket being USD 60,001–USD 80,000 (18.78%), and smaller groups reporting less than USD 20,000 (6.53%) or more than USD 160,000 (11.29%). Occupations were varied, with administrative/office staff comprising the largest group (17.28%), followed by “other” occupations (28.57%), and smaller proportions of sales/marketing professionals (9.93%), healthcare professionals (8.44%), and educators/teachers (6.80%).

Chi-square tests confirmed no significant differences in socio-demographic characteristics across the scenarios, ensuring the comparability of samples. For instance, gender (χ^2^ = 47.912, *p* > 0.05), age (χ^2^ = 106.180, *p* > 0.05), marital status (χ^2^ = 67.422, *p* > 0.05), income (χ^2^ = 115.931, *p* > 0.05), and occupation (χ^2^ = 133.041, *p* > 0.05) exhibited no substantial variation between groups. These results demonstrate that there were no significant differences in socio-demographic characteristics, which supports the validity of the experimental design and ensures that observed outcomes are attributable to the experimental manipulations rather than pre-existing differences in the sample.

### 4.2. Results of Overall Model

This section presents the results for the overall model, which examines how disease characteristics—spreading speed, severity, and vaccination requirements—affect travel intentions across the entire sample (Table 3). Spreading speed had a strong negative effect on travel intentions (F = 96.977, *p* < 0.01), with higher intentions for slow-spreading diseases (M = 4.55) compared to fast-spreading ones (M = 3.28). Disease severity also showed a significant effect (F = 172.656, *p* < 0.01), with mild diseases (M = 4.79) eliciting greater travel intentions than dangerous diseases (M = 3.14). Vaccination requirements had a weaker but still significant effect (F = 7.141, *p* < 0.01), with higher travel intentions when vaccination was not required (M = 4.08) compared to when it was required (M = 3.75).

**Figure 3:**
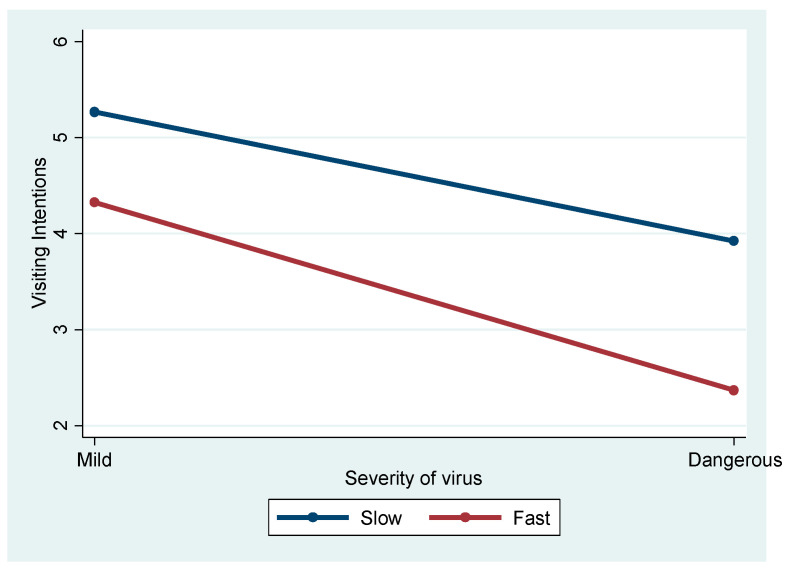
Interaction effect of spreading speed and severity of illness.

Figure 3 depicts the significant interaction between spreading speed and severity on intentions to travel (F = 5.805, *p* = 0.016). Post hoc comparisons revealed that travel intentions were highest for Slow Mild, which significantly differed from Fast Mild (Mean Difference = 0.931, *p* < 0.001) and Fast Dangerous (Mean Difference = 2.888, *p* < 0.001). The lowest travel intentions were observed for Fast Dangerous, which was significantly lower than Slow Mild (Mean Difference = −2.888, *p* < 0.001) and Slow Dangerous (Mean Difference = −1.536, *p* < 0.001). These findings indicate that individuals are most deterred by the combination of rapid disease transmission and severe health consequences, while slow-spreading, mild diseases have the least negative impact on travel intentions.

**Figure 4:**
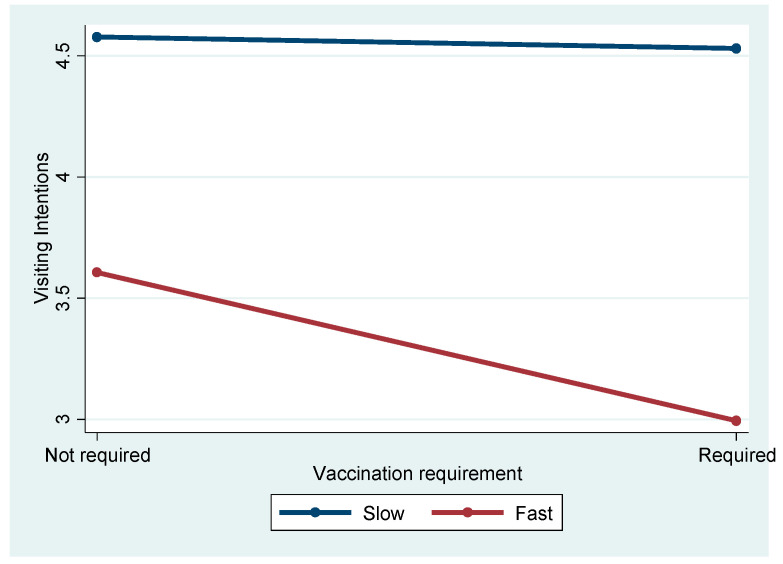
Interaction effect of spreading speed and vaccination requirement.

Figure 4 depicts the significant interaction effect between spreading speed and vaccination on travel intentions (F = 4.868, *p* = 0.028). Post hoc analyses showed no significant difference between Slow Not Required and Slow Required (Mean Difference = 0.057, *p* = 0.988). However, for fast-spreading diseases, vaccination requirements significantly reduced intentions, with Fast Required showing lower intentions than Fast Not Required (Mean Difference = −0.610, *p* = 0.003). Across spreading speeds, the negative effect of vaccination requirements was stronger under fast-spreading conditions (e.g., Slow Required vs. Fast Required, Mean Difference = −1.510, *p* < 0.001).

Among demographic variables, gender significantly influenced travel intentions (F = 4.160, *p* = 0.006), with men reporting higher intentions (M = 4.08) than women (M = 3.77). Having children in the household also increased travel intentions (F = 4.380, *p* = 0.037), with those with children reporting higher intentions (M = 4.07) compared to those without (M = 3.74). Other demographic factors, including marital status, age, and income, did not significantly affect travel intentions.

### 4.3. Results of Subsample: Virus Confined to the US

In this subsample analysis, the ANCOVA model explored how spreading speed, severity, and vaccination requirements influenced travel intentions when the virus was confined to the US (Table 4). Spreading speed had a significant negative effect on travel intentions (F = 53.817, *p* < 0.01). Respondents were more likely to travel when the disease was spreading slowly (M = 4.59) compared to quickly (M = 3.28). Similarly, severity had a strong impact (F = 79.866, *p* < 0.01). Mild diseases led to significantly higher travel intentions (M = 4.81) than dangerous diseases (M = 3.20). Vaccination requirements had a weaker but significant effect (F = 3.404, *p* < 0.10), with higher travel intentions reported when vaccination was not required (M = 4.09) compared to when it was mandatory (M = 3.76).

The interaction effects between the disease-related factors were not significant. The interactions between spreading speed and severity (F = 1.140, *p* = 0.286), spreading speed and vaccination requirements (F = 1.249, *p* = 0.265), and severity and vaccination requirements (F = 0.002, *p* = 0.966), as well as the three-way interaction (F = 0.547, *p* = 0.460), did not significantly influence travel intentions. These results indicate that the individual effects of spreading speed, severity, and vaccination requirements primarily drive travel intentions in the US context. Among demographic variables, only gender significantly influenced travel intentions (F = 3.572, *p* < 0.05). Other demographic factors, including marital status, the presence of children, age, and income, did not significantly affect travel intentions.

### 4.4. Results of Subsample: Virus with Global Spread

In this subsample analysis, the ANCOVA model assessed the impact of viral disease characteristics—spreading speed, severity, and vaccination requirements—on travel intentions to Orlando during a global viral disease outbreak (Table 5). The model was significant, explaining 32.6% of the variance in travel intentions (F = 9.01, *p* < 0.01). The spreading speed of the disease had a significant negative effect on travel intentions (F = 37.261, *p* < 0.01). Post hoc analyses showed that travel intentions were significantly lower for fast-spreading diseases (M = 3.31) compared to slow-spreading diseases (M = 4.48, *p* < 0.01). Similarly, disease severity also demonstrated a strong negative impact on travel intentions (F = 86.078, *p* < 0.01). Travel intentions declined substantially as disease severity increased from mild (M = 4.77) to dangerous (M = 3.05, *p* < 0.01). Vaccination requirements had a marginally significant effect (F = 3.173, *p* < 0.10), indicating that vaccination policies moderately influence travel intentions during a global viral outbreak. Post hoc results revealed that travel intentions were lower when vaccines were required (M = 3.74) compared to when they were not required (M = 4.06, *p* < 0.10).

**Figure 5:**
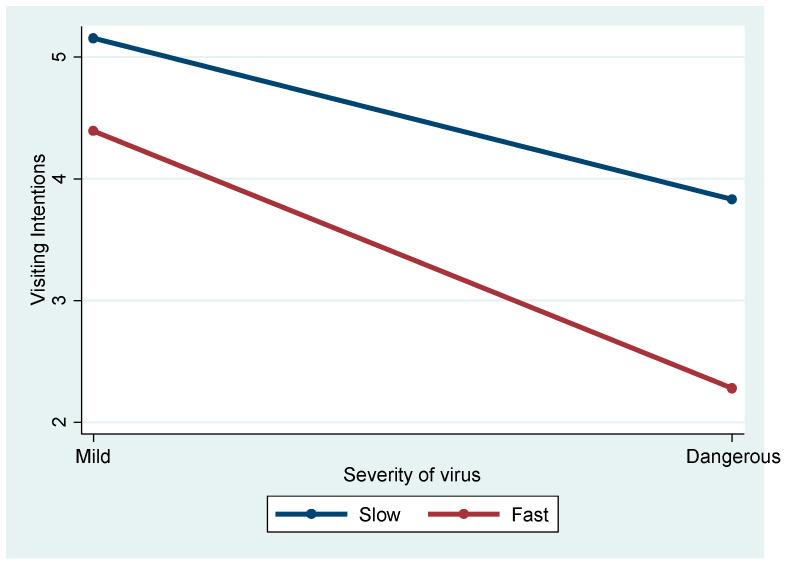
Interaction effect of spreading speed and severity of illness.

Figure 5 demonstrates a significant interaction effect between spreading speed and severity (F = 4.387, *p* < 0.05), suggesting that the combination of these factors influences travel intentions. Post hoc comparisons showed that travel intentions were particularly low for fast-spreading diseases that were also severe (M = 2.28), whereas slow-spreading mild diseases were associated with the highest travel intentions (M = 5.15, *p* < 0.01).

**Figure 6:**
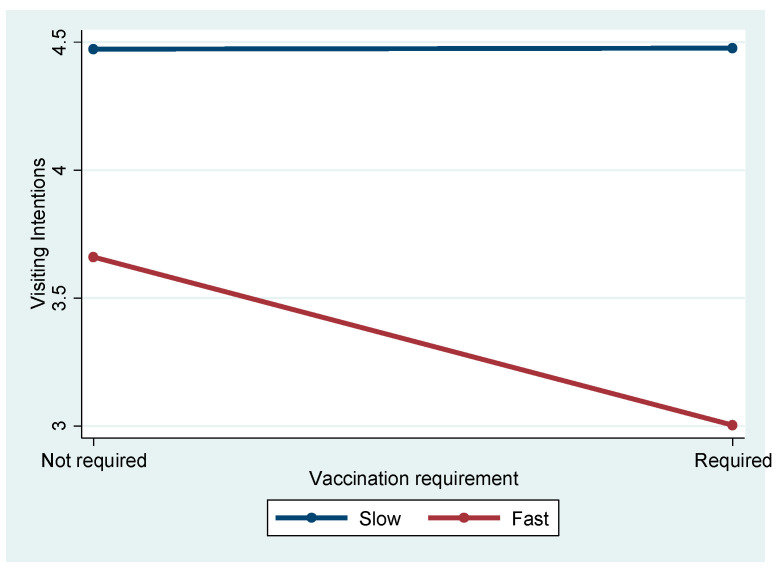
Interaction effect of spreading speed and vaccination requirement.

Figure 6 presents another marginally significant interaction effect observed between spreading speed and vaccination requirement (F = 3.120, *p* < 0.10). Post hoc analyses indicated that vaccination requirements had a stronger negative effect in fast-spreading scenarios than in slow-spreading ones. However, other interactions, including the three-way interaction among spreading speed, severity, and vaccination, were not significant (F = 0.25, *p* = 0.617). Among demographic factors, the presence of children in the household significantly influenced travel intentions (F = 3.643, *p* < 0.05). Respondents with children reported higher travel intentions (M = 4.13) compared to those without children (M = 3.68, *p* < 0.05). Other demographic variables, such as gender, marital status, age, and income, did not show significant effects on travel intentions in this subsample.

## 5. Conclusions

The results of the ANCOVA models provide important insights into how pandemic characteristics, such as disease-spreading speed, severity, and vaccination requirements, affect travel intentions. The overall model revealed that these factors accounted for 31.7% of the variance in travel decisions, highlighting their significant influence. Spreading speed emerged as a key factor, with travel intentions decreasing significantly as the speed of disease transmission increased (F = 96.977, *p* < 0.01). Disease severity had the most substantial negative impact on travel intentions (F = 172.656, *p* < 0.01), showing that more severe outbreaks greatly reduce the likelihood of travel. Although vaccination requirements were significant, they played a less dominant role (F = 7.141, *p* < 0.01), suggesting that while vaccination policies are important, they are secondary to the immediate threat posed by the speed and severity of the disease. This result is consistent with research on the hierarchy of perceived risks during health crises, where immediacy and severity often outweigh preventive measures such as vaccination.

The interaction effects revealed the complexity of travel decision-making during pandemics. The interaction between spreading speed and severity (F = 5.805, *p* < 0.05) suggests that these factors jointly amplify their impact on travel intentions, meaning that faster disease spread combined with greater severity significantly deters travel. Rapid transmission heightens uncertainty and fear, prompting travelers to avoid destinations, whereas high illness severity, such as mortality rates, leads to more drastic avoidance behavior. This interaction suggests that tourism destinations must respond with nuanced strategies, focusing on controlling transmission speed and addressing severity through clear communication and targeted safety measures. For instance, during a rapid yet mild outbreak, transparency and enhanced safety protocols can reassure travelers, whereas during severe outbreaks, emphasizing healthcare infrastructure and protective measures is essential [2,6,14]. These insights are vital for designing effective public health interventions and tourism recovery strategies, highlighting the need to consider both factors simultaneously when managing pandemic-related travel disruptions.

Similarly, the interaction between spreading speed and vaccination requirements (F = 4.868, *p* < 0.05) indicated that when diseases spread quickly, vaccination policies become more critical in travelers’ decision-making. The findings from this study provide valuable insights into how travelers’ decision-making is influenced by different pandemic attributes, specifically, the spreading speed of a disease and vaccination requirements. The significant interaction between spreading speed and vaccination requirements (F = 4.868, *p* < 0.05) suggests that when diseases spread rapidly, vaccination policies play a more critical role in shaping travelers’ decisions. This interaction indicates that travelers are more likely to consider the availability and importance of vaccination as a key protective measure when the disease is perceived to spread quickly. In high-speed transmission scenarios, travelers may view vaccination as a vital tool to protect themselves, particularly when they feel that the risk of exposure is imminent and unavoidable.

However, the lack of significance in interactions involving disease severity and vaccination requirements, as well as the three-way interaction, suggests that the combined influence of these factors does not significantly affect travel decisions in the same way. While disease severity and vaccination policies are individually important, they do not seem to amplify or compound one another when it comes to influencing travel behavior. This may imply that travelers tend to prioritize other factors, such as the speed at which a disease spreads, over the severity of the illness, or the potential preventive benefits of vaccination. In other words, when the risk of quick transmission is high, the urgency of prevention (such as vaccination) becomes more pronounced, but the severity of the disease itself does not significantly alter the decision-making process. For instance, during a rapid yet mild outbreak, transparency and enhanced safety protocols can reassure travelers, whereas during severe outbreaks, emphasizing healthcare infrastructure and protective measures becomes essential [2,6,14].

These findings align with models of individual risk assessment, which suggest that travelers weigh factors such as the speed of disease spread and perceived immediacy of risk more heavily than the severity of the disease or preventive measures, such as vaccination. In high-risk situations where rapid transmission is perceived, individuals may have less confidence in the effectiveness of vaccines, viewing them as insufficient in the short term, thus making them less central in their decision-making process. This can be seen as an adaptive response to the perceived urgency of the situation, where individuals focus on immediate risks and solutions rather than long-term preventive strategies.

When examining scenarios where the virus was confined to the US, similar patterns were observed, with the model explaining 32.6% of the variance in travel intentions. Spreading speed (F = 53.817, *p* < 0.01) and severity (F = 79.866, *p* < 0.01) were again the most influential factors, significantly deterring travel as perceived threat intensified. Vaccination requirements had a smaller yet still significant impact (F = 3.404, *p* < 0.10), reinforcing the idea that while vaccination policies matter, they are not as critical as the perceived speed and severity of the outbreak. In this context, demographic factors such as gender played a significant role (F = 3.572, *p* < 0.01), while marital status, presence of children, age, and income did not exhibit significant effects. This is consistent with findings from behavioral studies that show that gender often influences risk perception, with women typically exhibiting more cautious behavior in health-related decisions.

In the scenario where the virus spread worldwide, the model explained 32.6% of the variance in travel intention. Similar to the US-only subsample, the speed at which the disease spread (F = 37.261, *p* < 0.01) and its severity (F = 86.078, *p* < 0.01) had a significant negative impact on travel intentions. This highlights the strong deterrent effect of rapidly spreading and severe illness on international travel. Vaccination requirements also played a modest but significant role (F = 3.173, *p* < 0.10), showing that in a global context, vaccination policies become more relevant, although not as decisive as the immediate threat of the virus. Interaction effects, such as those between spreading speed and severity (F = 4.387, *p* < 0.05), suggest that these factors work together to heighten travelers’ reluctance, whereas other interactions, such as the three-way interaction, were not significant. The presence of children in the household significantly affected travel intentions (F = 3.643, *p* < 0.05), reinforcing that family related concerns can be critical in shaping travel behavior during global health crises.

These findings enhance our theoretical understanding of risk perception and decision-making during pandemics. Building on theories such as the Health Belief Model (HBM) and protection motivation theory, the results confirm that perceived risk—specifically, disease severity and speed—strongly influences travel intentions. This supports previous studies that highlight the central role of perceived severity in shaping cautious travel behavior [6,27]. While vaccination policies influence decision-making, their effect is less pronounced than the immediate threat posed by the disease. This highlights the complexity of travel behavior in health crises, where the most immediate and threatening factors often outweigh longer-term preventive measures, such as vaccination.

From a managerial perspective, these findings offer practical insights for the tourism and hospitality industries, particularly in high-tourism destinations such as Orlando. Understanding that disease spread speed and severity are the primary deterrents to travel underscores the importance of transparent communication and effective public health measures. Tourism managers should focus on providing real-time updates about disease spread and severity, as well as clear protocols to ensure traveler safety. Although vaccination requirements are important, they should be framed as part of a broader strategy to reduce risks, especially in the context of rapidly evolving health threats. Targeted marketing strategies can also be developed to address specific demographic concerns, such as promoting family friendly health protocols for travelers with children or creating gender-sensitive messaging that addresses varying levels of risk tolerance.

Our study revealed that certain demographic groups, such as women and families with children, exhibit heightened concerns about health risks and are more likely to alter or cancel travel plans during a pandemic. To target these groups more effectively, we suggest that tourism marketing efforts focus on gender-sensitive messaging and family-oriented health protocols. For women, messaging should emphasize the health and safety measures implemented at destinations, such as enhanced sanitation procedures, availability of health facilities, and precautions taken in accommodation and transport settings. This messaging can be reinforced through channels that are more likely to engage female travelers, such as social media platforms, women’s lifestyle blogs, and health-focused publications.

For families, marketing strategies should highlight family friendly features of destinations, such as childcare services, health safety measures for children, and the availability of family-centric activities that are low risk and socially distanced. Messaging could also reassure parents by emphasizing flexible booking policies and contingency plans in the case of health concerns, which might mitigate anxiety about potential cancellations or disruptions.

Furthermore, our data suggest that vaccination requirements, while significant, are not as decisive as the immediacy of disease threats. Therefore, tourism managers should consider positioning vaccination policies as part of a broader safety strategy rather than as a central solution. This could include promoting the availability of vaccines in key destinations and integrating vaccine-related messaging into general health communication. By making these adjustments, tourism stakeholders can better align their strategies with travelers’ concerns and ultimately enhance their confidence in traveling during a pandemic. We believe that these refined, data-driven recommendations provide more precise, actionable guidance for the tourism industry.

Tourism destinations can enhance resilience during health crises by prioritizing transparent communication and effective public health measures. Real-time updates about disease spread and clear protocols for traveler safety are crucial in maintaining confidence. These strategies should be communicated through diverse channels, such as official websites, mobile apps, and social media, ensuring accessibility for all travelers. Tailored marketing efforts, focusing on demographic groups like women and families, can address heightened health concerns. Gender-sensitive messaging should emphasize health measures for female travelers, while family-focused campaigns can highlight child-friendly activities and flexible booking options. These targeted approaches reassure vulnerable groups and encourage travel despite health uncertainties.

Additionally, vaccination requirements should be framed as part of a broader safety strategy rather than a central solution. Promoting the availability of vaccines alongside other health protocols, like sanitation and emergency measures, helps build trust in travel safety. Flexible booking policies and contingency plans are essential for mitigating concerns about potential cancellations. By adopting these proactive, data-driven strategies, tourism destinations can adapt to health challenges, ensuring continued traveler confidence and sustaining the tourism industry even during uncertain times.

Although this study provides valuable insights, its limitations must be acknowledged. Focusing on a single destination and relying on self-reported data may not fully capture the complexity of travel decision-making during pandemics. To address this, future research should include a wider array of variables, such as economic conditions, government policies, and broader contextual factors, and consider longitudinal approaches to tracking the evolution of travel intentions over time. Additionally, future studies should aim for a more diverse and inclusive sample encompassing a range of travel preferences, demographic profiles, and motivations. Incorporating more advanced analytical techniques could also help uncover deeper interactions among the variables at play. Despite these limitations, this study significantly contributes to our understanding of how pandemic characteristics influence travel behavior, offering both theoretical advancements and practical guidance for the tourism industry in managing future health crises.

## Figures and Tables

**Table 1:** Attribute details.

Attribute	Option	Description
Geographic Extension	Worldwide	The virus has spread globally.
	US-only	The virus is confined to the United States.
Disease Movement	Fast	The virus is spreading quickly. On average, each sick person passes it on to almost two others.
	Slow	The virus is spreading slowly. On average, each sick person passes it on to fewer than one other person.
Severity of Illness	Dangerous	The virus is extremely dangerous. Most people who catch it become very sick and require hospitalization.
	Mild	The virus is mild. Most people who catch it feel unwell briefly but recover quickly without medical help.
Vaccination Requirement	Required	Everyone is advised to get vaccinated to protect themselves and others.
	Not Required	Experts say vaccination is optional unless desired.

**Table 2:** Socio-demographic information of the sample.

Variable		Frequency	Percentage	χ^2^
Gender	Female	378	51.43%	47.912
	Male	348	47.35%	
	Prefer not to say	5	0.68%	
	Non-binary/third gender	4	0.54%	
Age	10s	15	2.04%	106.180
	20s	137	18.64%	
	30s	125	17.01%	
	40s	99	13.47%	
	50s	167	22.72%	
	60s	104	14.15%	
	70s	17	2.31%	
	80s	3	0.41%	
Marital Status	Married	324	44.08%	67.422
	Never Married	289	39.32%	
	Divorced	86	11.70%	
	Separated	14	1.90%	
	Widowed	13	1.77%	
	Prefer not to say	9	1.22%	
	Less than USD 20,000	48	6.53%	
Income	USD 20,001–USD 40,000	102	13.88%	115.931
	USD 40,001–USD 60,000	116	15.78%	
	USD 60,001–USD 80,000	138	18.78%	
	USD 80,001–USD 100,000	94	12.79%	
	USD 100,001–USD 120,000	60	8.16%	
	USD 120,001–USD 140,000	46	6.26%	
	USD 140,001–USD 160,000	48	6.53%	
	More than USD 160,000	83	11.29%	
Occupation	Administrative/Office Staff	127	17.28%	133.041
	Sales/Marketing	73	9.93%	
	Retired	63	8.57%	
	Healthcare Professional	62	8.44%	
	Educator/Teacher	50	6.80%	
	Engineer	46	6.26%	
	Unemployed	45	6.12%	
	Student	34	4.63%	
	Homemaker	25	3.40%	
	Other	210	28.57%	
Total		735	100.00%	

**Table 3:** ANCOVA results for the overall model.

Source	Partial SS	df	MS	F-Value	
Model	935.116	18	51.951	18.452	***
Spread	273.029	1	273.029	96.977	***
Severity	486.098	1	486.098	172.656	***
Vaccination	20.104	1	20.104	7.141	***
Spread × Severity	16.343	1	16.343	5.805	**
Spread × Vaccination	13.705	1	13.705	4.868	**
Severity × Vaccination	0.738	1	0.738	0.262	
Spread × Severity × Vaccination	1.348	1	1.348	0.479	
Gender	35.137	3	11.712	4.160	***
Marital Status	17.973	5	3.595	1.277	
Child	12.332	1	12.332	4.380	**
Age	6.120	1	6.120	2.174	
Income	0.482	1	0.482	0.171	
Residual	2015.831	716	2.815		
Total	2950.947	734	4.020		

Note: *** *p* < 0.01; ** *p* < 0.05. Number of observations: 735. R^2^ = 0.317.

**Table 4:** ANCOVA results for subsample (virus found in the US).

Source	Partial SS	df	MS	F-Value	
Model	488.834	18	27.157	9.732	***
Spread	150.172	1	150.172	53.817	***
Severity	222.858	1	222.858	79.866	***
Vaccination	9.499	1	9.499	3.404	*
Spread × Severity	3.182	1	3.182	1.140	
Spread × Vaccination	3.484	1	3.484	1.249	
Severity × Vaccination	0.005	1	0.005	0.002	
Spread × Severity × Vaccination	1.526	1	1.526	0.547	
Gender	29.906	3	9.969	3.572	**
Marital Status	17.455	5	3.491	1.251	
Child	1.341	1	1.341	0.481	
Age	5.900	1	5.900	2.115	
Income	1.980	1	1.980	0.709	
Residual	1010.132	362	2.790		
Total	1498.966	380	3.945		

Note: *** *p* < 0.01; ** *p* < 0.05; * *p* < 0.10. Number of observations: 381. R^2^ = 0.326.

**Table 5:** ANCOVA results for subsample (virus found worldwide).

Source	Partial SS	df	MS	F-Value	
Model	473.643	18	26.313	9.011	***
Spread	108.811	1	108.811	37.261	***
Severity	251.367	1	251.367	86.078	***
Vaccination	9.265	1	9.265	3.173	*
Spread × Severity	12.811	1	12.811	4.387	**
Spread × Vaccination	9.111	1	9.111	3.120	*
Severity × Vaccination	1.911	1	1.911	0.655	
Spread × Severity × Vaccination	0.731	1	0.731	0.250	
Gender	10.181	3	3.394	1.162	
Marital Status	7.270	5	1.454	0.498	
Child	10.639	1	10.639	3.643	*
Age	0.309	1	0.309	0.106	
Income	0.312	1	0.312	0.107	
Residual	978.278	335	2.920		
Total	1451.921	353	4.113		

Note: *** *p* < 0.01; ** *p* < 0.05; * *p* < 0.10. Number of observations: 354. R^2^ = 0.326.

## Data Availability

Data is available upon request.
